# Importance of untested infectious individuals for interventions to suppress COVID-19

**DOI:** 10.1038/s41598-021-00056-5

**Published:** 2021-10-20

**Authors:** Francisco J. Pérez-Reche, Ken J. Forbes, Norval J. C. Strachan

**Affiliations:** 1 School of Natural and Computing Sciences, University of Aberdeen, Old Aberdeen, Aberdeen, AB24 3UE Scotland, UK; 2 School of Medicine, Medical Sciences and Dentistry, University of Aberdeen, Foresterhill, Aberdeen, AB25 2ZD Scotland, UK

**Keywords:** Applied mathematics, Epidemiology

## Abstract

The impact of the extent of testing infectious individuals on suppression of COVID-19 is illustrated from the early stages of outbreaks in Germany, the Hubei province of China, Italy, Spain and the UK. The predicted percentage of untested infected individuals depends on the specific outbreak but we found that they typically represent 60–80% of all infected individuals during the early stages of the outbreaks. We propose that reducing the underlying transmission from untested cases is crucial to suppress the virus. This can be achieved through enhanced testing in combination with social distancing and other interventions that reduce transmission such as wearing face masks. Once transmission from silent carriers is kept under control by these means, the virus could have been fully suppressed through fast isolation and contact tracing of tested cases.

## Introduction

Daniel Defoe in “A Journal of the Plague Year” (1722) comments on the 1665 Great Plague of London that “. . . if all the infected persons were effectually shut in, no sound person could have been infected by them, because they could not have come near them. But the case was this (and I shall only touch it here): namely, that the infection was propagated insensibly, and by such persons as were not visibly infected, who neither knew whom they infected or who they were infected by.”^1^ COVID-19 is presenting similar problems today.

COVID-19 produced by the SARS-CoV-2 virus probably emerged in Wuhan, China in December 2019^2^ and has subsequently spread. By 10 April 2020 there were 1,521,252 confirmed cases and 92,798 deaths distributed in 213 countries^3^. One year later, there were around 135 million confirmed cases and almost 3 million deaths from 220 countries^4^. The worldwide burden of the disease is still very high despite significant efforts in many countries to suppress the spread of the virus. Until the end of 2020, when vaccines became available, efforts to suppress the virus focused on non-pharmaceutical interventions which ranged from handwashing and social distancing to contact tracing and more stringent measures such as isolation of infected individuals, banning of large gatherings or strict lockdowns^5–7^.

Optimising interventions to mitigate or suppress the burden of COVID-19 during an epidemic is a pressing global challenge due to significant uncertainties regarding the transmissibility of SARS-CoV-2 and other factors such as potentially large numbers of undocumented infections as well as political, social and economic considerations^8,9^. Underreported cases may include asymptomatic infected individuals^10–12^ and some that present symptoms but are not reported or are diagnosed with an alternative disease^13^. There is no consensus on the proportion of unreported cases and their potential impact on the spread of SARS-CoV-2. For instance, a World Health Organization report in February 2020 suggested that “the proportion of truly asymptomatic infections is unclear but appears to be relatively rare and does not appear to be a major driver of transmission”^14^.

Studies testing for SARS-CoV-2 infection in both symptomatic and asymptomatic individuals^10–12^, however, suggest that reported asymptomatic carriers can represent 50% or more of the cases at the time of testing. These may include true asymptomatic that remain asymptomatic throughout their infection and pre-symptomatic individuals that become symptomatic after testing. A comprehensive review suggests that around 20% of infected individuals are true asymptomatic^15^. Most countries test individuals when they have symptoms, and unreported infections are likely to include most of the asymptomatic individuals and a fraction of those with symptoms. Such asymptomatic individuals can act as silent carriers for SARS-CoV-2 and have been suggested as a key factor promoting the rapid spread of the virus^16^, similar to what has previously been observed with other infectious diseases^17^. An important question is to what extent isolation and contact tracing that has been implemented by many countries are effective in preventing the spread of SARS-CoV-2 if there is a significant proportion of infectious individuals that are not tested for it. On the positive side, if recovery from infection leads to immunity, one could hope that untested positive individuals could significantly contribute to the build-up of herd immunity in the population^18,19^. However, population-based sero-epidemiological studies suggest that the levels of population immunity reached through natural infection are far from that needed for herd immunity even in countries that have experienced the largest epidemics^20^.

Overall, the levels of underreporting of cases and the importance of such silent carriers on interventions for mitigation and suppression^21^ of the virus are not clear. Mathematical modelling has been very successful in epidemiology^22–24^ to devise and simulate suppression strategies and there is an ongoing effort to describe the dynamics of COVID-19 epidemics with mathematical models^5,16,19,21,25–37^. The effect of undocumented cases on the spread of the virus has been studied previously^16^, but the influence of such cases on control strategies has not been analysed. Asymptomatic individuals have been included in some models^5,28,30,33,34,37,38^ but the role of silent carriers in terms of untested cases is not fully understood.

Here we use mathematical models to quantify the levels of underreporting in several COVID-19 outbreaks and understand how this influences the spread of the virus and the efficacy of non-pharmaceutical measures to suppress it.

## Materials and methods

### Data

Our analysis uses data on numbers of infected and deceased individuals by country or region obtained from the Wolfram Data Repository^39^. We focus on the outbreaks in Germany, Hubei (China), Italy, Spain and United Kingdom (UK). All these countries have implemented social distance measures to control the spread of the virus. These measures involved global interventions that aim for a reduction of transmission at the population level (e.g., a lockdown) and local interventions involving isolation of infectious individuals and their contacts (see details in Additional file 1). Nationwide lockdowns were ordered in all the studied countries/regions to suppress the virus at the beginning of the outbreaks (see Sect. 1 in Additional file 1). These lockdowns were relaxed after periods that ranged between 56 days in Spain and around 100 days in the UK. Once the initial nationwide lockdowns were relaxed, all study countries kept stay-at-home policies which were particularly strict in China (see Sect. 2 in Additional file 1). In addition, all countries increased testing and contact tracing efforts. Among the European countries, Germany performed the largest number of tests per thousand people and had the lowest positive rate at the early stages of the epidemic (March and April 2020; see Figs. 4.2 and 5.2 in Additional file 1). From June 2020, the UK experienced the most prominent increase in testing efforts and registered the lowest positive rate (see Figs. 4.1 and 5.1 in Additional file 1). This was associated with the launching of test and trace schemes in the four nations of the UK during May 2020 (13 May in Wales^40^, 20 May in Northern Ireland^41^ and 28 May in England^42^ and Scotland^43^). We did not find detailed data on daily tests performed in China but according to the COVID-19 Government Response Tracker^44,45^, testing and contact tracing have remained at high levels in China since the early stages of the epidemic (see Sects. 3.1 and 4 in Additional file 1). In fact, only the measures in China were able to avoid a resurgence of the virus so far. The rest of the regions considered here had further waves of infection and all of them except Spain ordered further lockdowns at the end of 2020 and/or beginning of 2021 (see Sect. 1 in Additional file 1).

### Models

To describe the role of silent carriers in the spread of the virus, we used extensions of the SEIR model^16,23,25,29^ to include two types of infected individuals: tested (or reported) and untested (or unreported). The classification of infectious individuals into tested and untested classes intends to be in line with the available data which contains the number of tested cases. We remark that such classification does not make an explicit distinction between symptomatic and asymptomatic cases; in principle, both tested and untested classes may contain symptomatic and asymptomatic cases.

We propose two models: model 1 and model 2. Model 1 is a relatively simple extension of the SEIR model that we use to describe the early stages of epidemics in each of the regions. This model is fitted to data to estimate key epidemiological parameters and the proportion of tested and untested infectious individuals in each region (see parameter estimation methods below). Model 2 is a generalisation of model 1 to account for the isolation of infectious individuals and their contacts. The estimated parameters for model 1 are used to parameterise model 2. We now introduce both models, describe the main assumptions of the models and the methods for parameter estimation.

#### Model 1

**Figure 1:**
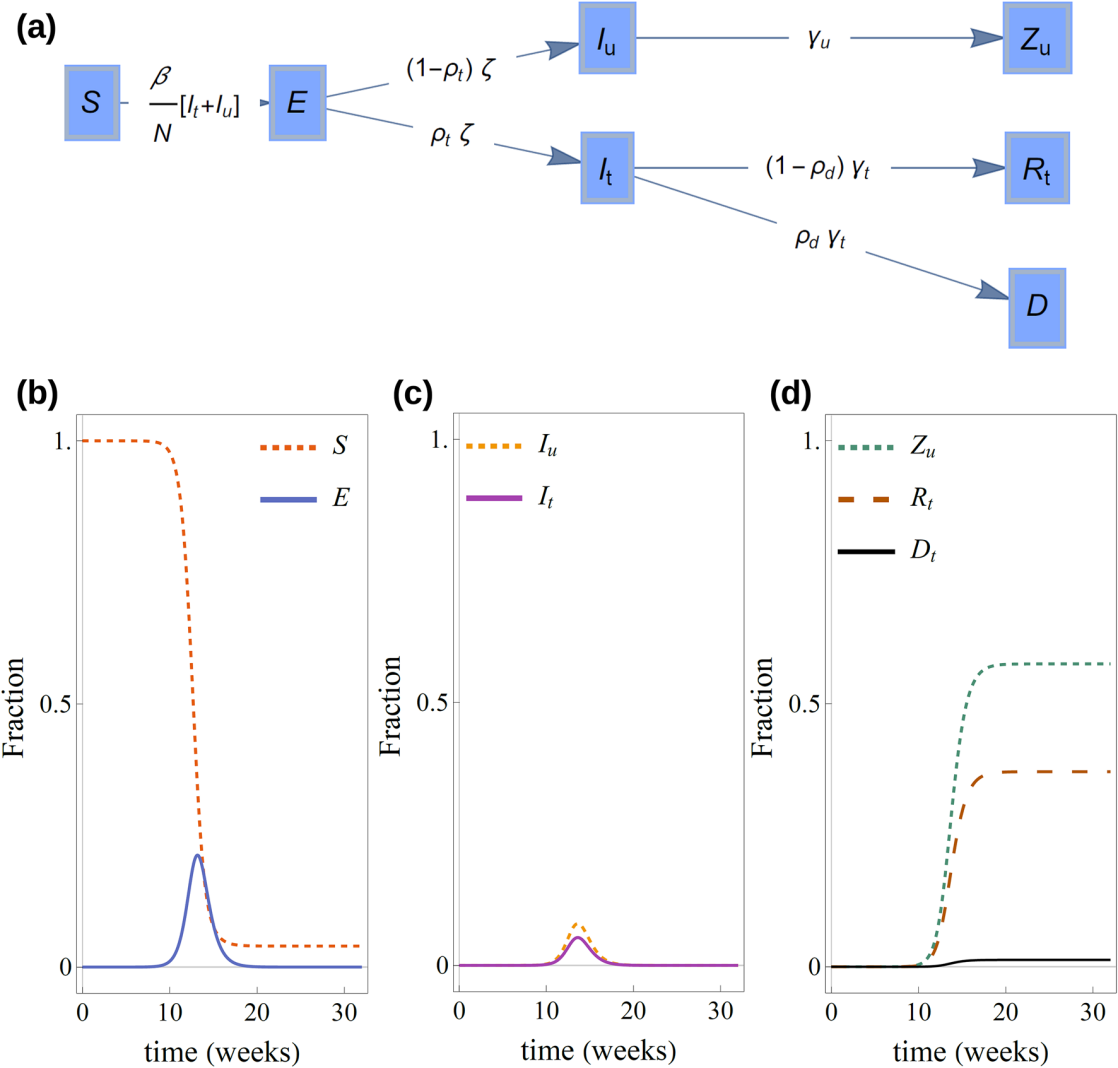
(**a**) Flow diagram for the compartment of model 1. Panels (**b–d**) show a typical time evolution of model variables. As the epidemic progresses, (**b**) the number of susceptible individuals decreases. The number of exposed individuals initially increases, reaches a peak and decreases at later stages of the epidemic. (**c**) The progression of the number of both tested and untested infected individuals also exhibits a peak. The decay of ${I}_{t}$ and ${I}_{u}$ after the peak induces a gradual weakening of the chain of transmission that leads to the end of the epidemic. (**d**) The number of tested and untested individuals that recover from infection or die increase monotonically during the epidemic.

Model 1 assumes that individuals can be in any of five compartments: Susceptible ($S$), exposed to the virus ($E$), tested infectious (${I}_{t}$), untested infectious (${I}_{u}$), recovered tested cases (${R}_{t}$), dead after testing positive ($D$) and untested infectious that recover or die (${Z}_{u}$). The flow between compartments is schematically shown in Fig. 1a.

The change of the number of individuals in each compartment is described by deterministic, continuous-time dynamics given by the following differential equations:

1 $$\begin{gathered} \dot{S} = - \beta \frac{{(I_{t} + I_{u} )S}}{N}, \hfill \\ \dot{E} = \beta \frac{{(I_{t} + I_{u} )S}}{N} - \zeta E, \hfill \\ \mathop {I_{t} }\limits = \zeta \rho_{t} E - \gamma_{t} I_{t} , \hfill \\ \mathop {I_{u} }\limits = \zeta (1 - \rho_{t} )E - \gamma_{u} I_{u} , \hfill \\ \mathop {R_{t} }\limits = \left( {1 - \rho_{d} } \right)\gamma_{t} I_{t} , \hfill \\ \mathop {Z_{u} }\limits = \gamma_{u} I_{u} , \hfill \\ \dot{D} = \rho_{d} \gamma_{t} I_{t} . \hfill \\ \end{gathered} $$

**Figure 2:**
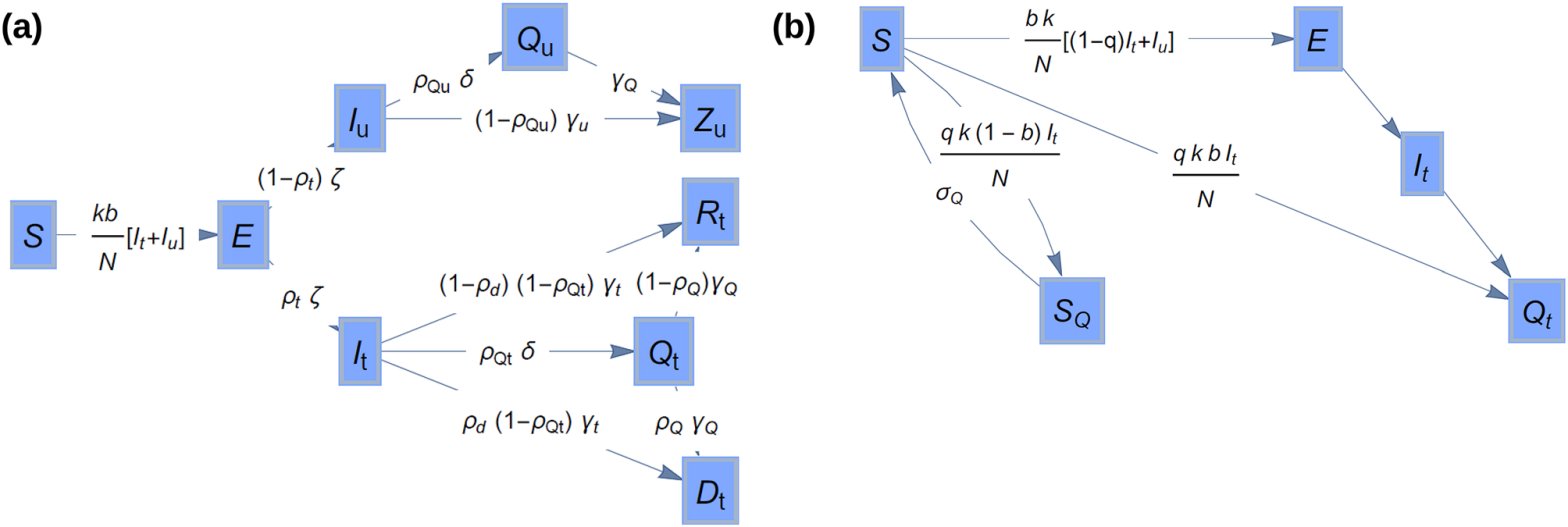
Model 2. (**a**) Extension of the flow diagram of model 1 (see Fig. 1) to incorporate a compartment ${Q}_{t}$ for isolation of tested infectious individuals and a compartment ${Q}_{u}$ for isolation of untested infectious individuals. (**b**) Flow diagram showing the implementation of contact tracing in model 2.

Here, $N$ is the population size (see Table S1 in Additional file 2). According to these equations, susceptible individuals (compartment $S$) become exposed to the virus (compartment $E$) at a rate $\beta ({I}_{t}+{I}_{u})/N$. Here, $\beta$ is the rate at which an infected individual transmits the infection to a susceptible individual and $({I}_{t}+{I}_{u})/N$ is the fraction of infectious individuals at time $t$. Exposed individuals remain in this state during a latent period ${\zeta }^{-1}$ after which they become infectious. Of those that are infectious, a fraction ${\rho }_{t}$ are tested for infection and move to the tested infected compartment ${I}_{t}$. The remaining fraction of infectious individuals, $1-{\rho }_{t}$, are not tested for infection and move to the untested infected compartment, ${I}_{u}$, after the incubation period. A fraction ${\rho }_{d}$ of infected individuals that were tested for infection, i.e. a fraction ${\rho }_{d}$ of ${I}_{t}$, die at a rate ${\gamma }_{t}$ and move to compartment, $D$. The rest of the tested cases move to the recovered compartment ${R}_{t}$. The fraction ${\rho }_{d}$ corresponds to the case fatality rate (CFR). The infection fatality rate (IFR) is the proportion of the total population that dies and can be determined from our models as $\mathrm{IFR}= {\rho }_{d}{\rho }_{t}$. Infected individuals in the untested compartment, ${I}_{u}$, move to the compartment ${Z}_{u}$. Assuming that recovered individuals are fully immune to infection, individuals in the compartment ${Z}_{u}$ are effectively removed from the epidemic. See a typical time evolution of the model variables in Fig. 2b-d.

The reproductive number corresponding to this model can be analytically calculated using the next generation method^24^ and conveniently expressed as the sum of two terms, ${\mathcal{R}}_{0}={\mathcal{R}}_{0}^{t}+{\mathcal{R}}_{0}^{u}$, giving the contribution of tested and untested individuals, respectively. The dependence of these contributions on the parameters of the model are given by the following expressions:

2 $$ {\mathcal{R}}_{0}^{t} = \frac{{\beta \rho_{t} }}{{\gamma_{t} }}, {\mathcal{R}}_{0}^{u} = \frac{{\beta \left( {1 - \rho_{t} } \right)}}{{\gamma_{u} }} $$

#### Model 2—Isolation and contact tracing

Model 2 is an extension of model 1 to study the effect of isolation of infected individuals and their contacts. This is achieved by adding two more compartments to model 1, ${Q}_{t}$ and ${Q}_{u}$, which contain isolated tested and untested infectious individuals, respectively (see Fig. 2a). The fraction of tested and untested infectious individuals who are isolated are denoted as ${\rho }_{Qt}$ and ${\rho }_{Qu}$, respectively. Both types of infectious individuals are assumed to become isolated at the same rate, $\delta$. Isolation of tested individuals is the most natural isolation strategy. Possible isolation of untested individuals, however, was incorporated to simulate scenarios in which some untested individuals may self-isolate without being tested if they exhibit symptoms or due to reasons that might not be linked to their infection (e.g. being advised to work from home or voluntarily trying to minimise contact). Contact tracing involves isolation of a fraction $q$ of susceptible individuals that were in contact with infected tested cases (see Fig. 2b). A fraction $b$ of these contacts would have acquired the virus through the contact (i.e. in the absence of contact tracing and isolation, they would have moved to the exposed compartment). These individuals are quarantined in the ${Q}_{t}$ compartment before they become infections and will eventually recover or die. The remaining contacts, i.e. a fraction $1-b$, remain susceptible after the interaction with the infected case and their isolation is represented by a compartment ${S}_{Q}$ where they remain for a period ${\sigma }_{Q}^{-1}$. After this period, such individuals remain susceptible and return to the $S$ compartment. With these assumptions, the number of individuals in each compartment evolve according to the following differential equations:

3 $$\begin{gathered} \dot{S} = - bk\frac{{(I_{t} + I_{u} )S}}{N} - q\left( {1 - b} \right)k\frac{{I_{t} S}}{N} + \sigma_{Q} S_{Q} , \hfill \\ \dot{E} = bk\frac{{(I_{t} + I_{u} )S}}{N} - \zeta E - qbk\frac{{I_{t} S}}{N}, \hfill \\ \mathop {I_{t} }\limits = \zeta \rho_{t} E - \left( {1 - \rho_{Qt} } \right)\gamma_{t} I_{t} - \rho_{Qt} \delta I_{t} , \hfill \\ \mathop {I_{u} }\limits = \zeta (1 - \rho_{t} )E - \left( {1 - \rho_{Qu} } \right)\gamma_{u} I_{u} - \rho_{Qu} \delta I_{u} , \hfill \\ \mathop {Q_{t} }\limits = \rho_{Qt} \delta I_{t} - \gamma_{Q} Q_{t} + qbk\frac{{I_{t} S}}{N}, \hfill \\ \mathop {Q_{u} }\limits = \rho_{Qu} \delta I_{u} - \gamma_{Q} Q_{u} , \hfill \\ \mathop {S_{Q} }\limits = q\left( {1 - b} \right)k\frac{{I_{t} S}}{N} - \sigma_{Q} S_{Q} , \hfill \\ \mathop {R_{t} }\limits = \left( {1 - \rho_{d} } \right)\left( {1 - \rho_{Qt} } \right)\gamma_{t} I_{t} + \left( {1 - \rho_{d} } \right)\gamma_{Q} Q_{t} , \hfill \\ \mathop {Z_{u} }\limits = \left( {1 - \rho_{Qu} } \right)\gamma_{u} I_{u} + \gamma_{Q} Q_{u} , \hfill \\ \dot{D} = \rho_{d} \left( {1 - \rho_{Qt} } \right)\gamma_{t} I_{t} + \rho_{d} \gamma_{Q} Q_{t} . \hfill \\ \end{gathered} $$

Here, $k$ is the number of contacts per unit time. The transmission rate is given by $\beta =kb$. Splitting the transmission rate into the number of contacts per unit time, $k$, and the fraction of successful transmissions in a contact, $b$, is necessary to model contact tracing^23^. Note that model 1 can be formally recovered from model 2 when the rates related to isolation and contact tracing are zero, i.e. when ${\rho }_{Qt}={\rho }_{Qu}={\gamma }_{Q}=q={\sigma }_{Q}=0$ in Eq. (3).

One can again use the next generation method^24^ to obtain an expression for the reproduction number of model 2 which can be expressed as the sum of two terms, ${\mathcal{R}}_{Q}={\mathcal{R}}_{Q}^{t}+{\mathcal{R}}_{Q}^{u}$, where

4 $$\begin{aligned} {\mathcal{R}}_{Q}^{t} &= \beta \frac{{\left( {1 - q} \right)\rho_{t} }}{{\gamma_{t} \left( {1 - \rho_{Qt} } \right) + \rho_{Qt} \delta }} ,\\{\mathcal{R}}_{Q}^{u} &= \beta \frac{{1 - \rho_{t} }}{{\gamma_{u} \left( {1 - \rho_{Qu} } \right) + \rho_{Qu} \delta }} . \\  \end{aligned}  $$

#### Control measures

Both model 1 and model 2 can be used to simulate measures such as a lockdown that achieve a reduction of transmission at the population level. This is simply implemented by reducing the intrinsic transmission rate $\beta$ by a factor $r\in [\mathrm{0,1}]$ (i.e. the reduced transmission is $\beta (1-r)$).

The effect of the initial lockdown ordered in all studied countries and subsequent relaxation was simulated with a piece-wise dependence of $\beta$ on time (see details in Sect. 1 of Additional file 3). The gradual increase in testing efforts, isolation of cases and contact tracing was simulated with model 2 assuming sigmoid growth functions for ${\rho }_{t}$, ${\rho }_{Qt}$ and $q$ (see details in Sect. 1 of Additional file 3).

#### Main assumptions of the models

Several simplifying assumptions were made to make the models operational and applicable to settings with limited data which are typical at early stages of epidemics. Here, we describe the main assumptions.

##### Homogeneous populations

The total number of individuals in each compartment of our models represent average values for the whole country. More precise descriptions at smaller scales such as geographical regions within countries or at the level of individuals would require accounting for spatial heterogeneity within the populations^31,46^ (e.g. cities and rural areas) as well as differences in both susceptibility and mortality across different age and vulnerability groups^47,48^ or topological heterogeneity of the network of contacts between individuals^49^.

Ignoring heterogeneities limits the ability of our models to identify specific ways to make interventions operational. For instance, reductions in transmission are treated at a generic level without specifying if they could be achieved by enhanced social distancing, school closure, etc. Accounting for such details would require using individual-based simulations^21^.

##### Deterministic dynamics

We focused on stages of epidemics in which the number of infectious individuals is large enough as for stochastic effects to be relatively unimportant on average^27^. Our models can be extended to incorporate stochasticity^50,51^. This would give a more accurate description of epidemics when SARS-CoV-2 has just invaded or at later stages when the number of infected individuals becomes very low.

##### All of the population at the start of the epidemic are susceptible

However, it may be that a proportion of the population are not susceptible for genetic reasons^52^ or due to cross immunity^53,54^.

##### Imported cases

We focused on epidemics that are at a stage in which imported cases are expected to play a secondary role relative to internal transmission. Accounting for imported cases is crucial, however, to prevent re-emergence of infection once an area has reached low numbers of infected individuals^55,56^. Imported cases could be included in our models in terms of an influx of infected individuals. In scenarios in which imported cases represent an important fraction of new infections, however, a stochastic version of the model would be more appropriate than the deterministic dynamics used here.

##### The transition times between compartments are exponentially distributed

This memory-less assumption is usual for classical compartmental models^23^. For COVID-19, however, transitions between compartments are better described in terms of gamma distributions^5,47^ and using models with memory would provide a more precise description of the dynamics^5,23,57^.

##### Tested and untested cases transmit infection at the same rate

The populations of tested and untested cases will typically consist of different proportions of symptomatic and asymptomatic cases. In spite of that, no statistical difference has been observed between the viral load of symptomatic and asymptomatic individuals^58,59^. It is therefore reasonable to assume that transmission is similar for symptomatic and asymptomatic individuals and therefore similar for tested and untested cases. Differences in overall transmission, however, are incorporated in the model through the assumption that recovery rates for tested and untested cases can be different.

##### Immunity follows after recovery from infection

Whether or not this is the case, and even if it is, the duration of the immunity is still unclear^60^. Our model could easily be extended to account for re-infections, should such data become available, and predictions might significantly change.

##### The latent and incubation periods coincide

We adopted a parsimonious approach which assumes that the latent period (i.e. the time between exposure to communicability) coincides with the incubation period (time between exposure and the appearance of symptoms)^25^. There is, however, a growing body of evidence for pre-symptomatic transmission^61–65^. There is the potential to incorporate this in our models by varying the incubation rate parameter, $\zeta$, or including a new compartment for pre-symptomatic infectious individuals^31^. We expect our predictions to be qualitatively independent of these details.

##### Reported deaths

We assume that the number of deaths in the datasets originates from individuals that were tested for infection.

##### Transmission in different regions

It is assumed that transmission of SARS-CoV-2 and the fraction of infectious cases are similar in different regions. This is reflected in the informative prior distributions used for ${\rho }_{d}$ and ${\mathcal{R}}_{0}$ in the parameter estimation procedure (see Table 1).

**Table 1:** Assumptions for the prior probability distribution of the estimated parameters.

Parameter	Prior	Support
Fraction tested infected,$\rho_{t}$	${\mathcal{U}}\left( {0,1} \right)$	Uninformative for $\rho_{t} \in \left[ {0,1} \right]$
Fraction of tested infected that die,$\rho_{d}$	${\mathcal{N}}\left( {0.034,0.01^{2} } \right)$	Mean set to the global estimate of WHO^85^
Recovery rate for untested infected,$\gamma_{t}$	${\mathcal{U}}\left( {0,0.4} \right)$	Range assumed from manual fit exploration (contains typical values for recovery rate^25,47^)
Recovery rate for untested infected,$\gamma_{u}$	${\mathcal{U}}\left( {0,0.4} \right)$	Similar assumptions as for $\gamma_{t}$
Reproduction number,${\mathcal{R}}_{0}$	${\mathcal{N}}\left( {4,1^{2} } \right)$	Estimates from Ref.^5^
Transmission rate,$\beta$	Derived from other parameters using Eq. (2)	
Number of exposed at the first data point,$E\left( 0 \right)$	$\ln E\left( 0 \right)\sim {\mathcal{N}}\left( {8,0.5^{2} } \right)$	Refs.^16,25^ and manual fit exploration

${\mathcal{N}}\left( {\mu ,\sigma^{2} } \right)$ denotes a normal distribution with mean $\mu$ and variance $\sigma^{2}$. ${\mathcal{U}}\left( {a,b} \right)$ denotes a uniform distribution in the interval $\left( {a,b} \right)$.

### Parameter estimation

We fitted the Model 1 to data. The value for the incubation rate was set to^48^ $\zeta =$ 1/5.2 days^-1^. The free parameters in our fits were the rate of transmission, $\beta$, proportion of infectious that were tested, ${\rho }_{t}$, proportion of tested infectious that die, ${\rho }_{d}$, rate to recovery of tested infectious individuals, ${\gamma }_{t},$ rate of recovery of untested infectious individuals, ${\gamma }_{u}$, and initial number of exposed individuals, $E(0)$. We denote the free parameters by a vector ${\varvec{\theta}}=\left\{\beta ,{\rho }_{t},{\rho }_{d},{\gamma }_{t},{\gamma }_{u},E\left(0\right)\right\}.$ The model was fit to the time series for the number of daily reported infected individuals and cumulative deaths, ${\mathcal{D}}_{\mathrm{obs}}= {\left\{{{i}_{\tau },d}_{\tau }\right\}}_{\tau =1}^{m}$, in a period of $m$ days in the early stages of epidemics (here, $\tau$ is used to denote discrete time in days). In particular, we used $m=15$ days since the first data point with a positive number of deaths (see Table S1 in Additional file 2). We used data at early stage of each outbreak to minimise the influence of suppression strategies on our parameter estimates.

Using data on deaths is important to obtain reliable descriptions of COVID-19 epidemics since data on deaths is likely to be more accurately recorded than data on infected and recovered individuals^5,19,38,66^. In addition to deaths, we can use data on infected individuals which is represented by the tested infectious compartment, ${I}_{t}$, in our models.

Our fitting procedure aims at calculating the posterior probability density function for the parameters given the data, $\pi ({\varvec{\theta}}|{\mathcal{D}}_{\mathrm{obs}})$. To this end, we use the procedure proposed in^51^ which can be regarded as an approximate Bayesian algorithm^67^. The posterior $\pi ({\varvec{\theta}}|{\mathcal{D}}_{\mathrm{obs}})$ is approximated by the empirical distribution of a set of 500 point estimates $\widehat{{\varvec{\theta}}}$ of the model parameters. A point estimate $\widehat{{\varvec{\theta}}}$ is obtained by simulating ${n}_{e}=3000$ epidemics with parameters sampled from a prior probability density $\widehat{\pi }({\varvec{\theta}})$. In each realization, a simulation of Model 1 produces deterministic evolution functions ${I}_{t}(t)$ and $D(t)$ for the number of tested cases and cumulative deaths. The functions ${I}_{t}(t)$ and $D(t)$ are used to build a random daily time series ${\mathcal{D}}_{\mathrm{sim}}({\varvec{\theta}})= {\left\{{i}_{\tau }^{\mathrm{sim}}\left({\varvec{\theta}}\right),{d}_{\tau }^{\mathrm{sim}}({\varvec{\theta}})\right\}}_{\tau =1}^{m}$, where ${i}_{\tau }^{\mathrm{sim}}$ and ${d}_{\tau }^{\mathrm{sim}}$ are, respectively, the number of tested infected and deaths predicted at day $\tau$. The point estimate $\widehat{{\varvec{\theta}}}$ is defined as the parameter vector corresponding to the realization that gives the closest prediction,${\mathcal{D}}_{\mathrm{sim}}$, to the observations, ${\mathcal{D}}_{\mathrm{obs}}$. More explicitly, the point estimate for the model parameters is given by

5 $$ \hat{\theta } = \arg \min_{\theta } \left\{ { \rho \left( {{\mathcal{D}}_{obs} |{\mathcal{D}}_{sim} \left( \theta \right)} \right)} \right\} , $$

where $\rho \left( {{\mathcal{D}}_{{{\text{obs}}}} {|}{\mathcal{D}}_{{{\text{sim}}}} \left( {\varvec{\theta}} \right)} \right)$ is a distance function. In particular, we used a weighted quadratic form for the distance:

6 $$ \rho \left( {{\mathcal{D}}_{obs} |{\mathcal{D}}_{sim} \left( \theta \right)} \right) = \mathop \sum \limits_{\tau = 1}^{m} \left[ {\frac{{\left( {i_{\tau } - i_{\tau }^{sim} \left( \theta \right)} \right)^{2} }}{{i_{\tau } }} + \frac{{\left( {d_{\tau } - d_{\tau }^{sim} \left( \theta \right)} \right)^{2} }}{{d_{\tau } }}} \right]. $$

Weighting by the observed values was used to account for the fact that the values taken by ${i}_{\tau }$ and ${d}_{\tau }$ differ by orders of magnitude. We checked, however, that fits with an unweighted distance give results that are statistically compatible with those reported in the main text. In addition, in the previous version of this work we obtained similar results by an approximate maximisation of a log-likelihood function in which ${i}_{\tau }^{\mathrm{sim}}({\varvec{\theta}})\sim \mathrm{Pois}({I}_{t}(\tau ))$ and ${d}_{\tau }^{\mathrm{sim}}({\varvec{\theta}})\sim \mathrm{Pois}(D(\tau ))$, i.e. the predicted number of tested infected and deaths were described as random variables obeying a Poisson distribution with mean ${I}_{t}(\tau )$ and $D(\tau )$, respectively^68^.

The prior probability density is defined as the product of priors for each parameter:

7 $$ \hat{\pi }\left( \theta \right) = \hat{\pi }\left( \beta \right)\hat{\pi }\left( {\rho_{t} } \right)\hat{\pi }\left( {\rho_{d} } \right)\hat{\pi }\left( {\gamma_{u} } \right)\hat{\pi }\left( {E\left( 0 \right)} \right) $$

The priors used in our fits are summarised in Table 1.

## Results

### Calibration of Model 1 at early stages of epidemics

**Figure 3:**
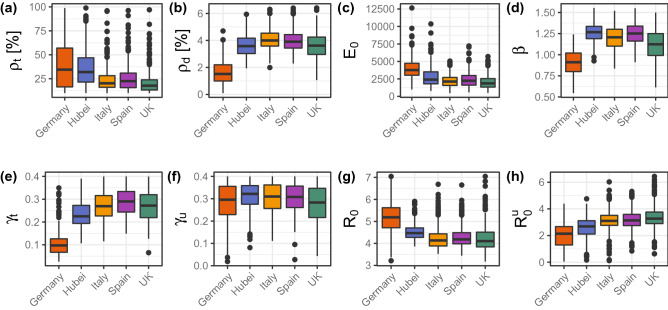
Boxplot statistics of the estimated parameters for model 1.

Figure 3 shows the estimates for the parameters of model 1 applied to the early stages of each outbreak (see numerical summary statistics in Additional file 2, Table S2). A comparison of the predicted trends and the data is shown in Additional file 2, Fig. S1.

The values for the testing coverage, ${\rho }_{t}$, reveal that during the early stage of outbreaks, Germany scored the highest in terms of testing for infection (34% [95% CI 10–96%]). Hubei follows Germany in terms of testing coverage, followed by Spain, Italy and the UK. Our prediction for Hubei (32% [95% CI 11–85%]) is statistically consistent with the 65% reporting estimated by Li et al.^16^ for China (not only Hubei) in the period 24 Jan–8 Feb 2020 which overlaps with the period 22 Jan–6 Feb 2020 used here. We note, however, that our estimate has a wide 95% CI and the comparison is not highly informative. The high testing percentage predicted for Germany agrees with the higher testing coverage at early stages of epidemics in this country (see Fig. 4.2 in Additional file 1). Taking the confidence intervals into account, we estimate that for each infected individual tested in the UK, there could have been between 1 and 9 untested infected individuals. At the other end of the testing spectrum, we estimate that for each infected individual tested in Germany, between 0.05 and 9 individuals might have not been tested at the beginning of the epidemic. A higher testing coverage for Germany is in qualitative agreement with estimates given elsewhere^28,38^. Our estimates for the reporting percentage, however, tend to be higher than those obtained by Jagodnik et al.^38^ and the differences we found between countries are not as extreme as those given by Chicchi et al.^28^.

From our estimates of ${\rho }_{t}$, we predict that 66% [95% CI 4–90%] of infected individuals were not tested for infection in Germany. Bearing in mind that 50% of infected individuals might be reported as asymptomatic at testing^10,12^, we conclude that untested cases in Germany would mostly correspond to asymptomatic cases at testing. In contrast, untested cases in other countries might include a significant number of symptomatic individuals in addition to asymptomatic cases. In particular, these may include infections of care home residents that are known to be underreported in many countries at early stages of epidemics^69^.

The proportion of tested infected individuals that die, ${\rho }_{d}$, is smaller for the outbreak in Germany than for the other outbreaks. This might be a combined effect of the fact that infected individuals in this country were relatively young at the beginning of the outbreak^70^ and the high testing rate. Indeed, the COVID-19 fatality rate is lower for the younger than for the elderly^47^ and, the higher the testing rate, the more individuals with mild or no symptoms will be included in the tested infected compartment of our model. The lower death rate of individuals with mild symptoms will lead to an effectively lower death rate for the whole set of infected individuals in this compartment. Accordingly, a lower value of ${\rho }_{d}$ does not necessarily mean a lower overall infection fatality rate. In fact, our estimates for $\mathrm{IFR}={\rho }_{t}{\rho }_{d}$ does not vary much among countries (between 0.5% [95% CI 0.1–1.1%] for Germany and 1.1% [95% CI 0.4–2.9%] for Hubei). These values are compatible with an IFR of 0.68% [95% CI 0.53–0.82%] reported for several studies in a systematic review^71^. Related to this, we found that the predicted fraction of deaths by the end of unmitigated epidemics is not too different for different countries either: 0.2% [95% CI 0.03–0.8%] for Germany, 0.8% [95% CI 0.3–3.6%] for Hubei, 0.7% [95% CI 0.3–2.1%] for Italy, 0.8% [95% CI 0.3–3.6%] for Spain and 0.6% [95% CI 0.2–2.3%] for the UK.

Our estimates for the time ${\gamma }_{t}^{-1}$ from reporting of infection to recovery or death are 3.4 [95% CI 2.4–6.0] days for Spain, 3.7 [95% CI 2.6–6.3] days for Italy, 3.7 [95% CI 2.6–6.5] days for UK, 4.4 [95% CI 2.7–7.1] days for Hubei and 10 [95% CI 3–26] days for Germany. The time for Hubei is consistent with estimates for China in other studies^16,35^. In general, the values we obtained are smaller than the infectious period (time from infection to death or recovery) reported elsewhere for COVID-19^5,47,72^. Our estimates thus probably reflect a reporting delay in all the studied outbreaks, in agreement with data on the onset of symptoms and reporting^70,73,74^. Under this hypothesis, our model predicts the smallest reporting delay for Germany, in agreement with a higher testing effort in this country at early stages of the epidemic.

The removal period for untested infected individuals, ${\gamma }_{u}^{-1}$, ranges between 3.1 [95% CI 2.5–6.1] days for Hubei and 3.5 [95% CI 2.5–8.0] days for the UK. Comparing with the reporting-to-recovery period ${\gamma }_{t}^{-1}$ and bearing in mind the reporting delays in all outbreaks, our estimates of ${\gamma }_{u}^{-1}$ suggest that untested individuals remain infectious for a shorter time than tested individuals.

We predict that the number of exposed individuals at the beginning of our simulations, $E(0)$, is of the order of several thousand for all the countries, in qualitative agreement with estimates of a previous study for China^16^.

The estimated reproduction number ${\mathcal{R}}_{0}$ is statistically similar in all the studied outbreaks. This reflects our prior assumption that transmission of SARS-CoV-2 is intrinsically similar in different regions. The transmission rate, $\beta$, was derived from the estimates of ${\rho }_{t}, {\gamma }_{t}, {\gamma }_{u}$ and ${\mathcal{R}}_{0}$ and it takes values that are around 1 for all countries.

### Suppression strategies

Suppression of the virus is achieved when the reproductive number is smaller than 1. In terms of model 2, the condition ${\mathcal{R}}_{Q}<1$ can be achieved in several different ways, i.e. there are many combinations of the parameters of the model that can lead to suppression of the virus (see Eq. 4). A simple suppression strategy consists in reducing the transmission rate $\beta$. We studied this strategy in April 2020 to make predictions related to the lockdowns that were active in Germany, Italy, Spain and the UK; the lockdown had just being lifted in Hubei^68^. We predicted that early reduction of transmission would delay the outbreaks but resurgence of infection was likely after relaxing the lockdowns unless transmission was kept reduced by a factor larger than 70% of its intrinsic values (see an update to these results in Sect. 2 of the Additional file 3). These results were prescient for Germany, Italy, Spain and the UK where further waves of infection occurred later in 2020.

In addition to nationwide lockdowns, all studied regions have implemented isolation and contact tracing programmes to suppress the virus. Following this, the reductions of $\beta$ mentioned above for scenarios without isolation and contact tracing should be interpreted as an effective measure of the reduction of transmission. Indeed, when isolation and contact tracing are taken into account, one needs a smaller reduction in $\beta$ to suppress the virus. Below we use model 2 to account for the combined effect of a reduced $\beta$ and enhanced testing, isolation and contact tracing in the design of suppression strategies bearing in mind the presence of silent carriers of the virus.

From Eq. (4) one can see that interventions that only isolate tested cases and their contacts can reduce the reproductive number ${\mathcal{R}}_{Q}^{t}$ associated with tested infected individuals. However, such strategy cannot suppress the virus since the reproductive number of untested cases is ${\mathcal{R}}_{Q}^{u}>1$ for all the studied epidemics (Fig. 3h). Ensuring that ${\mathcal{R}}_{Q}^{u}<1$ is then a crucial step for suppression of the virus. The reproduction number ${\mathcal{R}}_{Q}^{u}$ decreases with the testing rate, physical distancing and isolation of untested cases. Even in optimistic scenarios in which all tested cases would isolate in 0.5 days, we estimate that more than 25% of untested cases should isolate to suppress the virus. This is unlikely to occur by spontaneous isolation of untested cases (see Sect. 3 of the Additional file 3).

**Figure 4:**
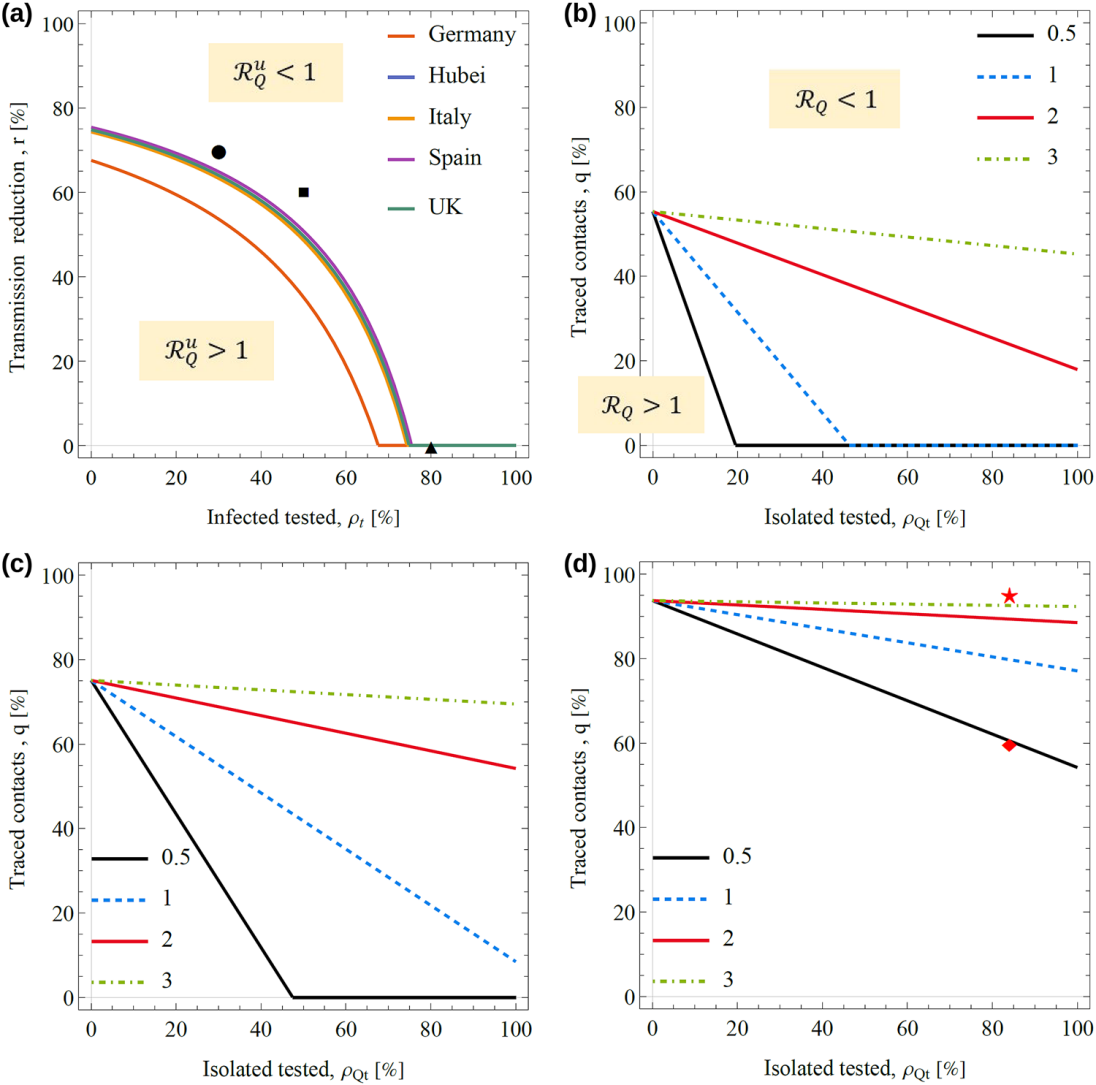
Suppression of the virus with physicals distance, enhanced testing, isolation and contact tracing. (**a**) Threshold lines for the reproduction number of untested cases as a function of the testing percentage $\rho_{t}$ and reduction of transmission, $r$. For a given country, ${\mathcal{R}}_{Q} < 1$ above the line and ${\mathcal{R}}_{Q} > 1$ below the line. The threshold lines for Italy and Hubei, Spain and UK are close to each other and hardly distinguishable in the plot. Taking the UK as an example, panels (**b**–**d**) show the threshold lines for suppression in terms of the isolation and contact tracing percentages of tested cases for different combinations of the testing percentage and reduction of transmission marked by the circle, square and triangle in panel (**a**). More explicitly, the parameters are $(\rho_{t} ,r) = \left( {30\% ,70\% } \right)$ in panel (**b**), $\left( {\rho_{t} ,r} \right) = \left( {50\% ,60\% } \right)$ in panel (**c**), and $\left( {\rho_{t} ,r} \right) = \left( {80\% ,0\% } \right)$ in panel (**d**). For a given panel, different lines indicate the threshold for different values of the time to isolation, $\delta^{ - 1}$, as marked by the legend. In panel (**d**), the diamond and star correspond to situations with $\left( {\rho_{Qt} ,q} \right) = \left( {84\% ,60\% } \right)$ and $\left( {\rho_{Qt} ,q} \right) = \left( {84\% ,95\% } \right)$, respectively, which were used to simulate the effect of enhanced isolation and contact tracing on the daily deaths in the UK (see Sect. 4 in Additional file 3). All lines show estimates based on the median of the model parameters.

We now focus on more feasible suppression approaches that combine enhanced testing and physical distancing with isolation and contact tracing of tested cases (i.e. no isolation is assumed for untested infectious individuals). The condition ${\mathcal{R}}_{Q}^{u}<1$ can be achieved through physical distancing and/or enhanced testing that are described by a reduction in $\beta$ and an increase in ${\rho }_{t}$, respectively. Figure 4a shows the threshold line corresponding to ${\mathcal{R}}_{Q}^{u}=1$ in the space $\left({\rho }_{t},r\right)$, where $r$ is the reduction factor of $\beta$. In particular, ${\mathcal{R}}_{Q}^{u}<1$ can be achieved without the need of physical distancing ($r=0$) if more than ~$80\%$ of infected individuals are tested. The condition ${\mathcal{R}}_{Q}^{u}<1$ could also be achieved without any testing (i.e. with ${\rho }_{t}=0$) if a severe reduction of transmission with $r\sim$ 80% is imposed. The later corresponds to a lockdown scenario studied above. Between these two extremes, the condition ${\mathcal{R}}_{Q}^{u}<1$ requires a combination of physical distancing and enhanced testing.

Once the condition ${\mathcal{R}}_{Q}^{u}<1$ is satisfied for untested cases, suppression can be achieved by reducing ${\mathcal{R}}_{Q}^{t}$ through isolation and contact tracing of tested cases. To illustrate this, we take the UK as an example and consider three scenarios with different levels of testing and social distancing.

Testing 30% of cases and reducing transmission by 70% through physical distancing leads to ${\mathcal{R}}_{Q}^{u}<1$ (circle in Fig. 4a) and suppression is possible if $\sim$ 20% of infected cases were isolated in ${\delta }^{-1}$ = 0.5 days (see black line in Fig. 4b). In this case, contact tracing would not be needed. For slower isolation of tested cases, however, contact tracing would be needed even if 100% of tested cases were isolated. This is illustrated in Fig. 4b by the lines corresponding to isolation in ${\delta }^{-1}$ = 2 or 3 days. Our results echo other studies in terms of the need to quickly isolate infected individuals^75^. In order to allow for a reasonable time between reporting of infection and isolation, carriers of the virus should be identified as early as possible, i.e. reporting delays should be minimised.

Suppression is also possible if 50% of cases are tested and strong physical distancing is imposed which keeps transmission reduced by a 60% (square in Fig. 4a). In this case, suppression without contact tracing is only possible for prompt isolation of tested cases (see black line in Fig. 4c). For slower isolation rates, a high coverage of contacts is needed even if all tested cases were isolated. For instance, if 100% of tested cases were isolated after 2 days, more than 60% of contacts should be traced to suppress the virus (see the red line in Fig. 4c).

In principle, mass testing and high compliance with isolation would make suppression possible without the need for physical distancing. For instance, suppression would be possible if > 80% of the cases were tested (triangle in Fig. 4a). As in the scenarios described above, high isolation and contact tracing would be needed to suppress the virus even if isolation is very fast (see Fig. 4d).

Detecting 80% of cases as assumed in the last scenario could be possible if, for instance, only truly asymptomatic cases escaped testing (around 20% of infected individuals are expected to be truly asymptomatic^15^). We tested the feasibility of this scenario for the second wave of infection observed in the UK after the relaxation of the initial lockdown. Stay-at-home and physical distancing policies remained active once the lockdown was relaxed in the UK. It is interesting, however, that the second wave of infection can be explained by model 2 in terms of a hypothetical scenario consisting of complete relaxation of the lockdown combined with enhanced testing and launching of test and trace schemes throughout the country. More explicitly, the scenario is as follows: Testing only misses asymptomatic cases (i.e.^15^ ${\rho }_{t}$ = 80%), 84% of those that tested positive self-isolate^76^ after 2 days of being tested^77^ and 60% of contacts are traced and isolated^78^ (this corresponds to the diamond in Fig. 4d that is below the red line, i.e. it is in the region with ${\mathcal{R}}_{Q}>1$). Full details of this analysis and a comparison of the predictions of model 2 with data are given in Sect. 4 of the Additional file 3. We checked that a similar scenario in which 95% of contacts are traced instead of 60% leads to suppression of the virus (see the star symbol in Fig. 4d which is above the red line where ${\mathcal{R}}_{Q}<1$).

Even though a scenario that assumes a complete relaxation of the lockdown can reproduce the data in the UK, a scenario with a partial relaxation of the lockdown is more realistic since stay-at-home and physical distancing policies remained active after the relaxation of the lockdown. This would indicate that the levels of testing, isolation and contact tracing were probably less effective than those used above for complete relaxation of the lockdown. For example, we checked that a scenario with post-lockdown transmission reduced by 20% and an isolation rate of 20% also captures the observed trend for the number of dealy deaths after the lockdown (see Fig. S4(c) in Sect. 4 of the Additional file 3). In general, there can be many combinations of the parameters of model 2 leading to a reasonable description of the data. In spite of that, our results suggest that high levels of testing, isolation and contact tracing could suppress the virus even if 20% of cases were asymptomatic and not tested for infection.

## Conclusions

The main aim of our modelling work is to contribute to the understanding of the epidemiological patterns of SARS-Cov-2. The models should be viewed as a general guide of how the outbreak and interventions may play out rather than as an exact representation of COVID-19 epidemics. In spite of our simplifying assumptions, there are two main implications from the models which are relevant for health policy in dealing with the outbreak.

The first, involves the existence of a significant proportion of cases that are not tested and act as silent carriers of the infection. We found that the predicted percentage of untested infected individuals at the early stages of epidemics may have represented 60–80% of the cases. The specific percentage depends on the country and we found the lowest proportion of unreported cases in Germany. The levels of underreporting are expected to have gradually decreased during the course of the studied epidemics since testing capacity increased in all the studied regions. It is likely, however, that underreporting remains significant due to asymptomatic individuals or cases with mild symptoms.

Our model predicts that a resurgence of the virus was likely after relaxing the initial lockdown in 2020 in all the studied regions. This would be similar to second waves of infection observed in the 1918 influenza epidemics^79^. In fact, we made this prediction in April 2020 when lockdowns were still active in the studied European countries and it had just been relaxed in Hubei^68^. This prediction was confirmed by the resurgence of cases observed in European countries in August 2020^80^.

The second implication involves the finding that unreported cases play an important role in the control of COVID-19 epidemics. In particular, unreported cases act as silent carriers and control strategies need to account for them or be prone to the risk of re-emergence or ineffective suppression of spread. For instance, we predict that isolation and contact tracing of tested cases can have a limited impact on the suppression of spread unless the underlying transmission of silent carriers is suppressed. The latter can be achieved by combining physical distancing and thorough testing of case contacts. Related to this, we found that physical distance might not be essential in an ideal scenario in which testing only misses 20% of cases (e.g. asymptomatic), there is a high adherence to self-isolation policies and contact tracing is highly effective. In the absence of pharmaceutical interventions, however, a certain level of physical distance is likely to be necessary for suppression. To summarise, in line with previous work^19,81^ and our predictions in April 2020, we suggest that widespread testing combined with contact tracing^26,27^, isolation of infected individuals and social distancing are necessary to suppress SARS-CoV-2 using non-pharmaceutical interventions without severe lockdowns.

Vaccinations are now available and our models could be extended to study the combined effect of vaccination and non-pharmaceutical interventions on epidemics while accounting for underreporting. Information on the effects of vaccines is still limited but it is expected that they will reduce the risk of individuals becoming infected and will protect against COVID-19 symptoms and severe illness^82–84^. Gradual reductions of the risk of infection and risk of death can be readily simulated by assuming gradually decreasing the transmission rate ($\beta$) and proportion of infected individuals that die (${\rho }_{\mathrm{d}}$). A reduction in $\beta$ leads to a reduction of the reproduction number of both tested and untested infectious individuals and this aids suppression of the virus. Enhanced protection against symptoms plays a less clear role in the suppression and would require a more detailed analysis. Indeed, protection against symptoms might lead to an increased proportion of silent carriers of the virus. This might lead to an increase in the reproduction number that might ultimately reduce some of the benefits of the vaccine. Based on early evidence that vaccines reduce the risk of serious illness^82^, prioritising the vaccination of the most vulnerable individuals is crucial to make sure that a potential resurge of transmission associated with silent carriers occurs when those individuals have been already vaccinated. In addition to that, keeping high levels of testing and isolation of cases will be crucial to prevent potential negative effects associated with unreported cases.

## Supplementary Information

Supplementary Information 1.

Supplementary Information 2.

Supplementary Information 3.

## Data Availability

All data used in this work are available from the cited sources. The models were analysed implemented in Mathematica. A notebook that retrieves the data and runs the calculations can be downloaded from https://doi.org/10.6084/m9.figshare.14636199. Point estimates of the parameters of model 1 and an R script to generate Fig. 3 can also be downloaded from this link.
