# Supplementary material for: Importance of untested infectious individuals for interventions to suppress COVID-19
Source: Sci Rep. 2021 Oct 20;11:20728. doi: 10.1038/s41598-021-00056-5 (PMC8528842; doi:10.1038/s41598-021-00056-5)
Supplement: Supplementary file 1 — Supplementary Information 1. [file 41598_2021_56_MOESM1_ESM.pdf]

Importance of untested infectious individuals for interventions to suppress COVID-19 Additional file 1: Measures to mitigate/suppress the virus

Francisco J. Pérez – Reche, Ken J. Forbes and Norval J. C. Strachan

This file shows the time dependence of measures taken by the studied countries to test for the virus and mitigate/suppress it. More specifically, information is given for the lockdown periods, stay at home policy, testing (positive rate and number of tests) and contact tracing policy.

1. Lockdown periods

Table of lockdown periods in the studied countries and regions.

**Country/ Region**	**Lockdown periods**
Germany	22/03/20 - 11/05/20, 02/11/20 - 19/04/21
Hubei	Wuhan:<br>23/01/20 – 25/03/20,<br>Hubei province:<br>13/02/20 – 25/03/20
Italy	09/3/20 – 18/05/20, 24/12/20 – 06/01/21, 15/03/21 – 30/04/21
Spain	14/03/20 – 09/05/20
UK	England:<br>23/03/20 – 04/07/20, 05/11/20 – 02/12/20, 05/01/21 – 29/03/21<br>Northern Ireland:<br>23/03/20 – 03/07/20, 27/11/20 – 11/12/20, 26/12/20 – 12/04/21<br>Scotland:<br>23/03/20 – 29/06/20, 26/12/20 – 02/04/21<br>Wales:<br>23/03/20 – 13/07/20, 23/10/20 – 09/11/20, 20/12/20 – 13/03/21

The following figure shows the timeline plot for lockdown periods in the studied outbreaks. Time intervals indicate periods under lockdown. For Hubei, the time interval corresponds to the lockdown in Wuhan. For the UK, intervals correspond to lockdown dates in England, the most populated region in the country. Solid circles for Germany, Italy, Spain and UK show the date of the first death.

Out[ ]=

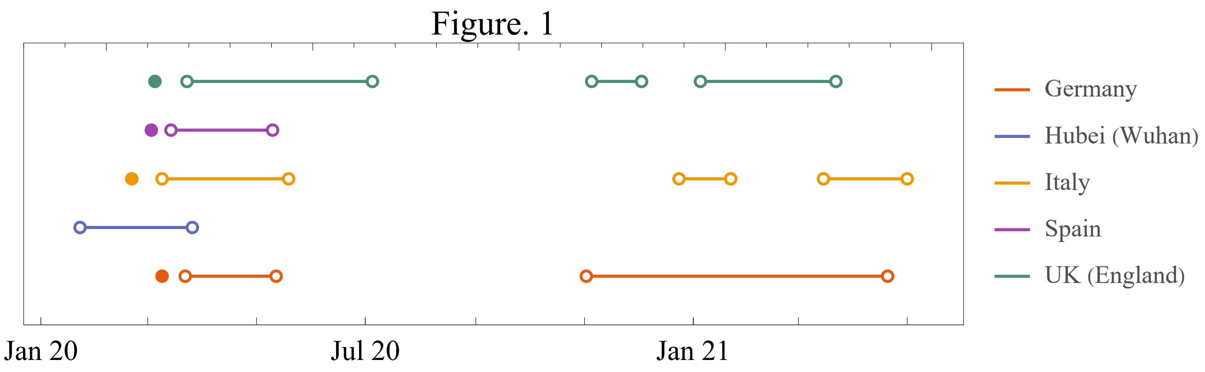

The data was obtained from https://en.wikipedia.org/wiki/COVID-19_lockdowns for all countries except for the Hubei province which was obtained from https://en.wikipedia.org/wiki/COVID-19_-pandemic_in_Hubei.

2. Stay at home

Policies on stay-at-home requirements or household lockdown.

Countries are grouped into four categories:
0 - No measures
1 - recommend not leaving house
2 - require not leaving house with exceptions for daily exercise, grocery shopping, and ‘essential’ trips
3 - Require not leaving house with minimal exceptions (e.g. allowed to leave only once every few days, or only one person can leave at a time, etc.)

Data source:
COVID-19 Government Response Tracker
https://ourworldindata.org/covid-stay-home-restrictions

Time series for the stay-at-home policy level in the studied countries (no data was available for Hubei province and data for China is presented instead):

Out[ ]=

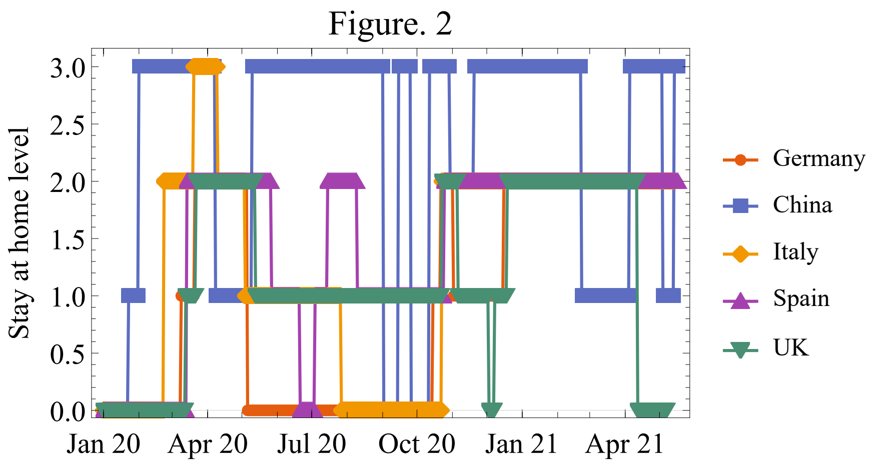

Table of the mean and standard deviation of the stay at home level during the period shown in the figure:

Out[ ]//TableForm=

Country	Level
Germany	1.0 ± 0.9
China	2.3 ± 1.1
Italy	1.3 ± 0.9
Spain	1.4 ± 0.8
United Kingdom	1.2 ± 0.7

3. Testing

3.1. Testing policy

Government policies on testing for COVID-19. Note that this relates to PCR testing for the virus only; it does not include non-PCR, antibody testing.

Countries are grouped into four categories:
0 – No testing policy
1 – Only those who both (a) have symptoms AND (b) meet specific criteria (eg key workers, admitted to hospital, came into contact with a known case, returned from overseas)
2 – testing of anyone showing COVID-19 symptoms
3 – open public testing (eg “drive through” testing available to asymptomatic people)

Data source:
COVID-19 Government Response Tracker
https://ourworldindata.org/covid-testing-contact-tracing#testing-policy

Time series for the testing policy level in the studied countries (no data was available for Hubei province and data for China is presented instead):

Out[∘]=

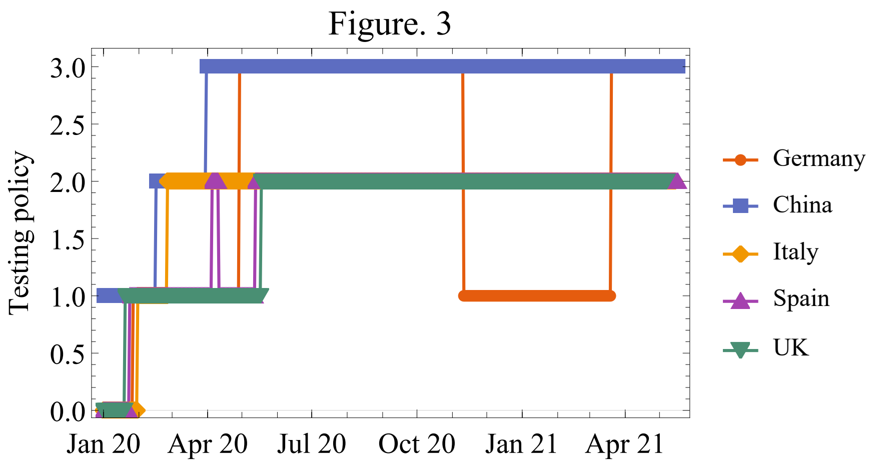

Table of the mean and standard deviation of the testing policy level during the period shown in the figure:

Out[∘]//TableForm=

Country	Level
Germany	1.0 ± 0.9
China	2.3 ± 1.1
Italy	1.3 ± 0.9
Spain	1.4 ± 0.8
United Kingdom	1.2 ± 0.7

3.2. Daily tests

Seven-day rolling average of the number of daily tests per thousand people.

Data source: https://ourworldindata.org/coronavirus-testing#testing-for-covid-19-background-the-our-world-in-data-covid-19-testing-dataset
Information is not available for China.

Out[∘]=

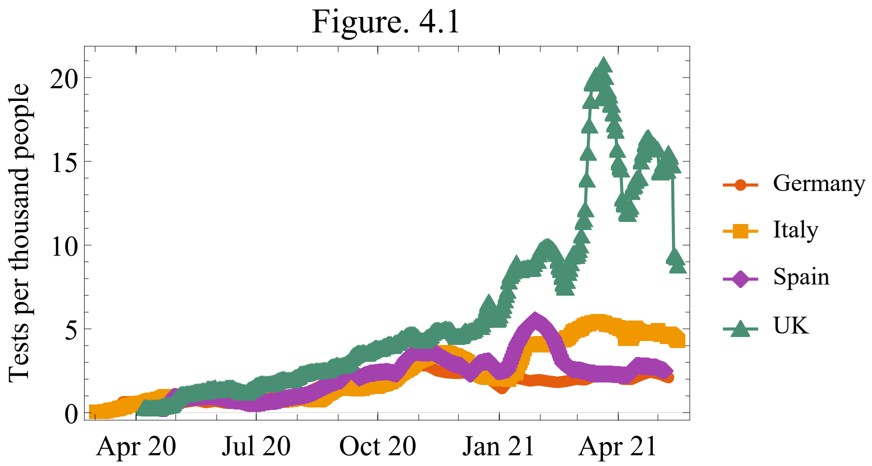

Detail of the daily tests per thousand people at early stages of the outbreaks:

Out[ ]=

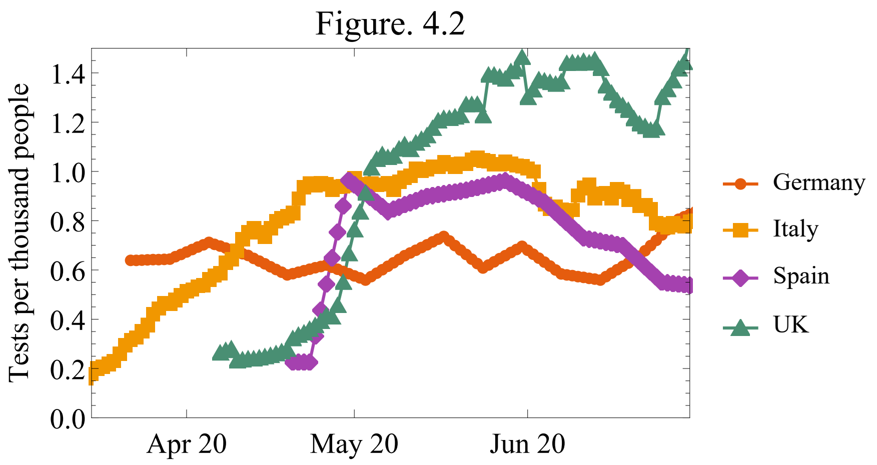

3.3. Positive rate

Percentage of tests returning a positive result.

Data source: https://ourworldindata.org/coronavirus-testing#testing-for-covid-19-background-the-our-world-in-data-covid-19-testing-dataset
Information is not available for China.

Out[ ]=

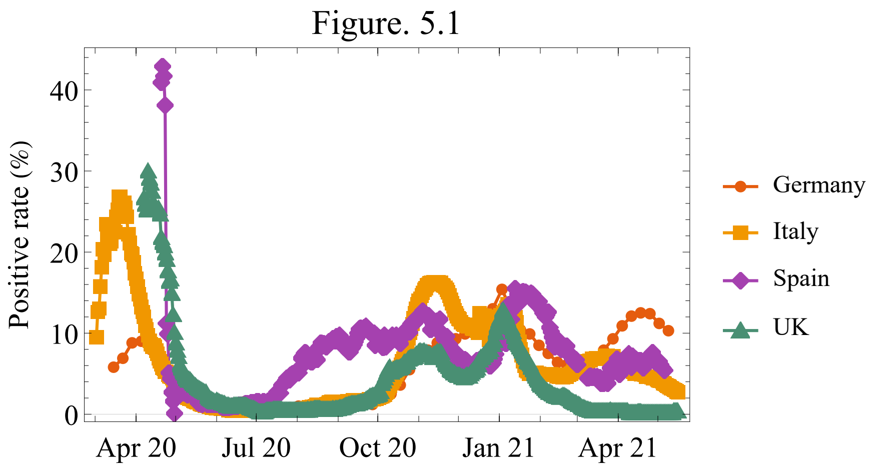

Detail of the positive rate at early stages of the outbreaks:

Out[∘]=

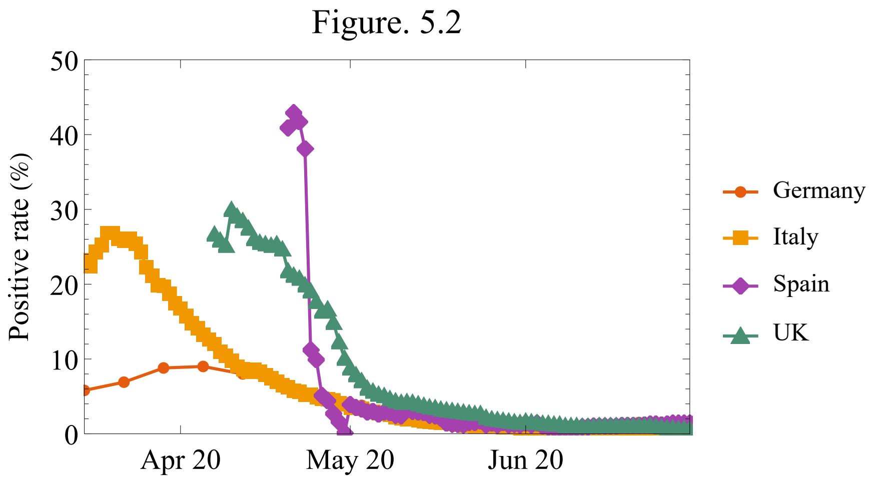

4. Contact tracing

Contact tracing coverage levels:

0 - No contact tracing

1 - Limited contact tracing - not done for all cases

2 - Comprehensive contact tracing - done for all cases

Data source:

COVID-19 Government Response Tracker

https://ourworldindata.org/covid-testing-contact-tracing#contact-tracing

Time series for the contact tracing coverage level in the studied countries (no data was available for Hubei province and data for China is presented instead):

Out[∘]=

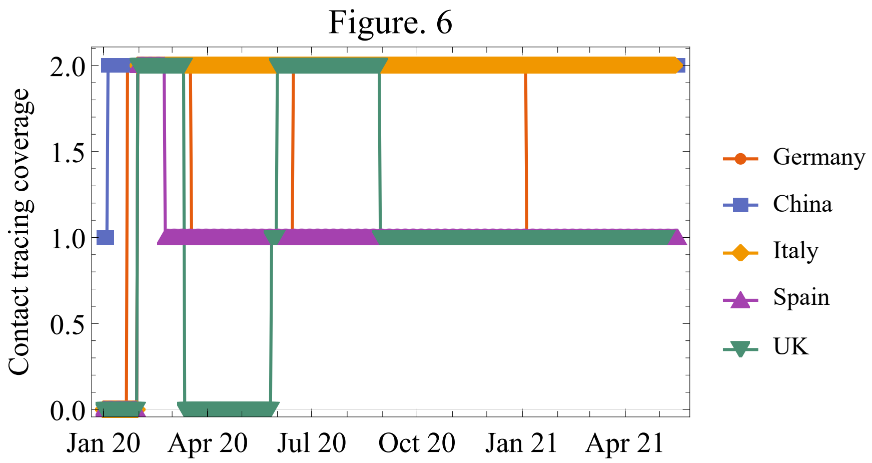

Table of the mean and standard deviation of the contact tracing coverage level during the period shown in the figure:

Out[ ]//TableForm=

Country	Level
Germany	1.5 ± 0.6
China	1.99 ± 0.09
Italy	1.9 ± 0.5
Spain	0.99 ± 0.33
United Kingdom	1.1 ± 0.7
