# Supplementary material for: Importance of untested infectious individuals for interventions to suppress COVID-19
Source: Sci Rep. 2021 Oct 20;11:20728. doi: 10.1038/s41598-021-00056-5 (PMC8528842; doi:10.1038/s41598-021-00056-5)
Supplement: Supplementary file 2 — Supplementary Information 2. [file 41598_2021_56_MOESM2_ESM.docx]

**Importance of untested infectious individuals for interventions to suppress COVID-19**

**Additional file 2: Supplementary figures and tables**

Francisco J. Pérez-Reche, Ken J. Forbes and Norval J. C. Strachan

Table S1. Details on the first day used to calibrate the models, number of deaths by that day and population of each country/region.

| **Country/Region** | **First day considered** | **Number of deaths on first day considered** | **Population, *N*** |
| --- | --- | --- | --- |
| Germany | 09/03/20 | 2 | 82,114,224 |
| Hubei | 22/01/20 | 17 | 58,160,000 |
| Italy | 21/02/20 | 1 | 59,359,900 |
| Spain | 03/03/20 | 1 | 46,354,321 |
| UK | 05/03/20 | 1 | 66,181,585 |

Table S2. Estimates of the model parameters given in terms of the 2.5% percentile, median and 97.5% percentile. $\beta$ is the transmission rate, $\rho_{t}$ is the proportion of tested cases (in percentage), $\rho_{d}$ is the proportion of tested cases that die (in percentage), $\gamma_{t}$ is the rate of recovery of tested cases, $\gamma_{u}$ is the rate of recovery of untested cases, $E(0)$ is the initial number of exposed individuals .


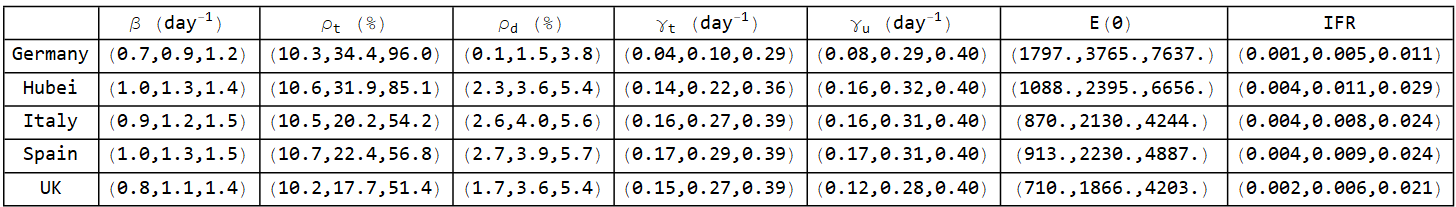


from R0_with_contact_tracing.nb with snipping tool

| **(a)** Germany | **(b)** Hubei | **(c)** Italy |
| --- | --- | --- |
|  |  |  |
| **(d)** Spain | **(e)** UK |  |
|  |  |  |

Figure S1. Number of tested infected at a given time ($I_{t}$, red) and cumulative dead ($D_{t}$ black/grey) individuals registered in (a) Germany, (b) Hubei, (c) Italy, (d) Spain and (e) UK. Symbols show the data. Shaded regions show the 90% confidence interval of model predictions at any given time. Time given in days since the first day with a positive number of deaths in the datasets. Logarithmic scale is used in the vertical axis of each plot.
