# Supplementary material for: Importance of untested infectious individuals for interventions to suppress COVID-19
Source: Sci Rep. 2021 Oct 20;11:20728. doi: 10.1038/s41598-021-00056-5 (PMC8528842; doi:10.1038/s41598-021-00056-5)
Supplement: Supplementary file 3 — Supplementary Information 3. [file 41598_2021_56_MOESM3_ESM.docx]

**Importance of untested infectious individuals for interventions to suppress COVID-19**

**Additional file: Additional results on suppression strategies**

Francisco J. Pérez-Reche, Ken J. Forbes and Norval J. C. Strachan

Table of Contents

[1. Time-dependent control measures 1](#_Toc73711852)

[2. Suppression by reducing the transmission rate at the population level – Early predictions 2](#_Toc73711853)

[3. Effect of isolating untested infected individuals 5](#_Toc73711854)

[4. Suppression by enhancing testing, isolation and contact tracing 5](#_Toc73711855)

Here, we present data and model predictions for daily deaths and include additional results on scenarios for control of the spread of the virus. In Section 1, we describe the implementation of time-dependent control measures in our models to simulate measures taken in the analysed outbreaks (periods with enhanced physical distance and gradual increase of testing, case isolation and contact tracing). Section 2 present predictions for the effect of a lockdown followed by a period in which the lockdown is fully or partially relaxed. This scenario simulates the situation after the initial lockdown implemented in all the studied regions in 2020 was relaxed. Section 3 discusses suppression based on isolation of both tested and untested infectious individuals. Finally, section 4 presents scenarios based on model 2 in which an initial lockdown is combined with gradually increased testing, isolation of tested infectious individuals and their contacts.

# Time-dependent control measures

To simulate the lockdown periods initially ordered in the studied countries, we assume that the transmission is reduced by a factor $r_{1}$ during an interval of time $[t_{a},t_{b})$ and this is followed by a relaxation to a new situation with transmission reduced by a factor $r_{2}\leq r_{1}$. More explicitly, we assume the following piece-wise dependence of the transmission rate on time:

|  | $\beta_{r}\left( t \right)=\left\{ \begin{aligned} \begin{matrix} \beta, & t<t_{a} \end{matrix} \\ \begin{matrix} \beta\left( 1-r_{1} \right), & t_{a}\leq t<t_{b} \end{matrix} \\ \begin{matrix} \beta\left( 1-r_{2} \right), & t\geq t_{b}. \end{matrix} \end{aligned} \right.$ | (S1) |
| --- | --- | --- |

In principle, one could extend this piece-wise form to simulate further lockdowns. However, we restrict our analysis to the first ordered lockdown and its subsequent relaxation.

A gradual increase in the testing efforts is simulated by a time-dependent fraction of tested cases given by

|  | $\rho_{t}\left( t \right)=\rho_{t}^{\min}+\left( \rho_{t}^{\max}-\rho_{t}^{\min} \right)f\left( \frac{t-t_{\rho}}{\tau_{\rho}} \right) ,$ | (S2) |
| --- | --- | --- |

where $f\left( x \right)=\left( 1+\exp\left( -x \right) \right)^{-1}$ is a sigmoid function. Accordingly, $\rho_{t}(t)$ grows from $\rho_{t}^{\min}$ and $\rho_{t}^{\max}$ in a characteristic time of the order of $\tau_{\rho}$ centred around the time $t_{\rho}$. For the predicted infection fatality rate (IFR) to remain unchanged as the fraction of tested cases changes, the fraction of tested infected individuals that die should be expressed as $\rho_{d}=\mathrm{IFR}/\rho_{t}(t)$ in the equations of Model 2 (Eq. (4) of the main text).

A gradual increase in isolation of positive cases and contact tracing efforts is described by assuming that $\rho_{Qt}$ and $q$ grow from zero to maximum values $\rho_{Qt}^{\max}$ and $q^{\max}$, respectively. More explicitly, we assume a sigmoid growth for both quantities:

|  | $\rho_{Qt}\left( t \right)=\rho_{Qt}^{\max}f\left( \frac{t-t_{q}}{\tau_{q}} \right) \mathrm{and} q\left( t \right)=q^{\max}f\left( \frac{t-t_{q}}{\tau_{q}} \right).$ | (S3) |
| --- | --- | --- |

For simplicity, here we assume that the characteristic times $t_{q}$ and $\tau_{q}$ are identical for both $\rho_{Qt}$ and $q$.

# Suppression by reducing the transmission rate at the population level – Early predictions

In this section, we present predictions for suppression of the virus by simply reducing physical distance, i.e. by reducing the transmission rate $\beta$. Since the isolation of cases and contact tracing are neglected, predictions are based on model 1. As illustrated in Figure S1(a), an outbreak can be significantly delayed if the transmission rate is reduced at an early stage in the epidemic, in agreement with other works^1^. The predicted number of deaths by the end of the epidemic, however, only reduces significantly when $\beta$ is reduced by a factor close to $r=1-1/\mathcal{R}_{0}$ to ensure an early eradication of the infection^2^. Based on our estimated values for $\mathcal{R}_{0}$, this requires reducing the transmission rate by more than 70% in all of the studied outbreaks. This is illustrated in Figure S1(b) for the UK. As can be seen, the number of deaths would only reduce significantly if the number of contacts were reduced by approximately 80%.

The model predicts that lockdowns ordered at early stages of the pandemic lead to significant reductions in the number of daily deaths in all the studied countries. This agrees with the data (see the upper panels in Figure S2). Table S1 shows values of the parameters used to simulate the lockdown (see Eq.(S1)). These parameters were estimated through manual exploration of the fit of the model to the data (see Section 2.7 in the Mathematica notebook available from <https://figshare.com/s/69d9aedcbf8b312b4c46>). Fits were only attempted for the first 35 weeks (trying to fit the model for later times would require at least accounting for further lockdowns). We estimate that initial lockdowns reduced the transmission by more than 80% in all the regions we analysed (see $r_{1}$ values in Table S1). This ensures a reproduction number $\mathcal{R}_{0}<1$. Irrespective of the effectiveness of the lockdowns, however, the model predicts a resurgence of the epidemics if lockdowns were completely lifted (see the marked increase in the number of deaths predicted when the lockdowns are relaxed, i.e. when $r_{2}=0$ in the upper panels of Figure S2). By tuning the value of $r_{2}$ to fit the increase in the number of daily deaths (middle row in Figure S2), we conclude that transmission of infection remained reduced by at least 55% after relaxation of the initial lockdown in all the studied outbreaks (see column $r_{2}$ (observed) in Table S1). The reduction level was insufficient to prevent a second wave of infection in all studied regions except Hubei. In particular, we predict that transmission must have been reduced by at least 80% after the lockdown in Hubei. This was probably not only achieved through a reduction of $\beta$ but rather through a combination of reduced $\beta$ and increased testing, isolation and contact tracing. In the lower row of Figure S2 we show scenarios in which a resurgence in the number of deaths could have been avoided in all other countries if the transmission had been kept reduced by 70 – 80% of its intrinsic value (see the used values in the column $r_{2}$ (suppress)).

| **(a)** | **(b)** |
| --- | --- |
|  |  |

Figure S1. Predicted effect of reducing the transmission rate $\beta$ on the outbreak in the UK in a hypothetical scenario in which the intervention was applied from the beginning of the epidemic and kept active until the end. (a) Proportion of tested infectious individuals, $I_{t}$, as a function of time if no interventions are implemented (red) or if the transmission rate is reduced by 30% (green) or 60% (brown) since the beginning of the epidemic. (b) Effect of transmission reduction, $r$, on the fraction of the susceptible population that die, $D$, during the epidemic. The line gives the median and the shading represents the 95% confidence interval of the model predictions.

| **(a)** Germany | **(b)** Hubei, China | **(c)** Italy | **(d)** Spain | **(e)** UK |
| --- | --- | --- | --- | --- |
|  |  |  |  |  |
|  |  |  |  |  |
|  |  |  |  |  |

Figure S2. Data for daily deaths (symbols) and predictions of model 1 with a lockdown period followed by several different strategies (i.e. different reduced transmissions $r_{2}$): (upper row) full relaxation with $r_{2}=0$, (middle row) $r_{2}$ set to reproduce the observed daily deaths after relaxation and (bottom row) $r_{2}$ kept at a high enough value to keep a low number of deaths. Values for the parameters $t_{a}$, $t_{b}$, $r_{1}$ and $r_{2}$ are given in Table S1. The thick grey line gives the median of the predictions and grey shading represent the 95% confidence interval of the model estimate.

Table S1. Value of the parameters for the simulations shown in Figure S2 for a lockdown followed by an exit strategy with a reduction in transmission (see Eq. (1)).

| **Region** | $\boldsymbol{t}_{\boldsymbol{a}}$ **(weeks)** | $\boldsymbol{t}_{\boldsymbol{b}}$ **(weeks)** | $\boldsymbol{r}_{\boldsymbol{1}}$ | $\boldsymbol{r}_{\boldsymbol{2}}$ **(observed)** | $\boldsymbol{r}_{\boldsymbol{2}}$ **(suppress)** |
| --- | --- | --- | --- | --- | --- |
| Germany | 3.5 | 25 | 0.92 | 0.55 | 0.80 |
| Hubei | 2.0 | 12 | 0.90 | 0.80 | 0.80 |
| Italy | 3.7 | 24 | 0.82 | 0.70 | 0.75 |
| Spain | 3.7 | 20 | 0.85 | 0.60 | 0.73 |
| UK | 3.5 | 24 | 0.82 | 0.60 | 0.73 |

# Effect of isolating untested infected individuals

From a theoretical perspective, the condition $\mathcal{R}_{Q}^{u}<1$ for the reproduction number of untested cases can be achieved by isolating such cases. Combining this with isolation of tested individuals could lead to eradication of the disease in all the studied outbreaks provided the period $\delta^{-1}$ from reporting to isolation is short enough. Figure S3 shows the estimated boundaries that separate the eradication region ($\mathcal{R}_{Q}<1$) from the epidemic region ($\mathcal{R}_{Q}>1$) as a function of the fractions of isolated tested and untested cases for a short isolation delay of $\delta^{-1}$= 0.5 days. For instance, a hypothetical intervention in the UK in which all tested infected individuals were isolated in $\delta^{-1}$= 0.5 days could suppress the spread of the virus if 40% of untested infected were isolated at the same rate. For Germany, the percentage of untested individuals that should be isolated to ensure eradication in this scenario is lower (~25%) since our estimates suggest that testing was already faster and more effective than in other countries at early stages of epidemics.


$$\mathcal{R}_{Q}>1$$

$$\mathcal{R}_{Q}<1$$

Figure S3. Interventions in which a fraction $\rho_{Qt}$ of tested cases and a fraction $\rho_{Qu}$of untested cases are isolated in an average time of $\delta^{-1}$= 0.5 days. The lines separate the regions in the space $(\rho_{Qt},\rho_{Qu})$ where eradication occurs (above the line for a given country) from the regions where the epidemic grows (below the line). The lines are based on the median of $\mathcal{R}_{0Q}$.

# Suppression by enhancing testing, isolation and contact tracing

This section shows predictions for the number of daily deaths in the UK based on model 2. Predictions are based on a lockdown ordered $t_{a}=3.5$ weeks after the first death was registered, followed by a relaxation at $t_{b}=24$ weeks. The lockdown implemented in the model is longer than the official lockdown in the UK that lasted for ~15 weeks. We hypothesise that stay-at-home and self-isolation policies effectively prolonged the effects of the lockdown after relaxation. In addition to the lockdown, we assume a gradual increase of testing, isolation and contact tracing that were implemented as described in Section 1, with the parameters values and justification given in Table S2. Figures S4(a) and (b) show compare the predictions of the model with the data in scenarios in which the lockdown is fully relaxed (i.e. $r_{2}=0$). The difference between panels (a) and (b) is the level of contact tracing ($q^{\max}= 60\%$ and $q^{\max}= 95\%$, respectively) reached after the program is launched at the end of May 2020 (week $t_{q}=12$ in the plots). Figures S4(c) shows the predictions for a scenario in which the lockdown is partially relaxed to a reduced transmission with $r_{2}=20\%$ and the isolation level is $\rho_{Qt}^{\max}=20\%$, i.e. smaller than in panels (a) and (c) where $\rho_{Qt}^{\max}=84\%$.

Table S2. Parameters used in model 2 to simulate a lockdown followed by a relaxation of restrictions combined with a gradual increase of testing, isolation and contact tracing (see model predictions and data in Figure S4).

| **Parameter** | **Value or distribution** | **Support** |
| --- | --- | --- |
| $r_{1}$ | 0.82 | Estimated through manual exploration of the fit of the model to daily deaths. |
| $r_{2}$ | 0 in Figure S4 (a) and (b), 0.2 in Figure S4 (c) | Set to test various scenarios in different panels of Figure S4. |
| $t_{a},t_{b}$ (weeks) | 3.5, 24 | Manual exploration of the the fit of the model to daily deaths in the first 24 week sof the outbreak. The same values as for model 1 (see Table S1). |
| $\rho_{t}^{\min}$ | Point estimates from fit of model 1 |  |
| $\rho_{t}^{\max}$ | $\mathcal{N(}{0.8,0.025}^{2})$ | Based on estimates from Buitrago-Garcia et al.^3^ |
| $t_{\rho}$ (weeks) | 24 | Assumed in our scenario. |
| $\tau_{\rho}$ (weeks) | 2 | Assumed in our scenario. |
| $\rho_{Qt}^{\max}$ | 0.84 in Figure S4(a) and (b), 0.2 in Figure S4(c) | Data from^4^. |
| $q^{\max}$ | 0.6 in Figure S4(a) and (c), 0.95 in Figure S4(b) | From the fraction of traceable contacts estimated in^5^. |
| $t_{q}$ (weeks) | 12 | Corresponds to the end of May when the test and trace schemes were launched in the UK (see Data section in Materials and Methods of the main text). |
| $\tau_{q}$ (weeks) | 1 | Assumed in our scenario (but tested for compatibility with the data). |
| $\delta^{-1}$ (days) | 2 | From turnaround times in England, 2020^6^. |
| $\gamma_{Q}^{-1}$ (days) | 10 | UK policy for self-isolation^7^. |
| $\sigma_{Q}^{-1}$ (days) | 10 | UK policy for self-isolation^8^. |

Figure S4. Predictions of model 2 for the number of daily deaths in the UK in three different scenarios with an initial lockdown that is relaxed concurrently with a gradual increase of testing, isolation and contact tracing. Circles show the data, the thick grey line gives the median of the predictions and grey shading represent the 95% confidence interval of the model estimate. The model parameters are summarised in Table S2. The only parameters that differ for different panels are $r_{2}$, $\rho_{Qt}^{\max}$ and $q^{\max}$: (a) $r_{2}=0$, $\rho_{Qt}^{\max}=0.84$, $q^{\max}=0.6$, (b) $r_{2}=0$, $\rho_{Qt}^{\max}=0.84$,$q^{\max}=0.95$ and (c) $r_{2}=0.2$, $\rho_{Qt}^{\max}=0.2$,$q^{\max}=0.6$.

# References

1. Ferguson, N. M., Laydon, D., Nedjati-Gilani, G. & et al. *Impact of non-pharmaceutical interventions (NPIs) to reduce COVID-19 mortality and healthcare demand*. https://doi.org/10.25561/77482 (2020).

2. Anderson, R. M. & May, R. M. *Infectious diseases of humans: dynamics and control*. (Oxford University Press, 1991).

3. Buitrago-Garcia, D. *et al.* Occurrence and transmission potential of asymptomatic and presymptomatic SARS-CoV-2 infections: A living systematic review and meta-analysis. *PLOS Medicine* **17**, e1003346 (2020).

4. Coronavirus and self-isolation after testing positive in England - Office for National Statistics. https://www.ons.gov.uk/peoplepopulationandcommunity/healthandsocialcare/healthandwellbeing/bulletins/coronavirusandselfisolationaftertestingpositiveinengland/1februaryto13february2021.

5. Keeling, M. J., Hollingsworth, T. D. & Read, J. M. Efficacy of contact tracing for the containment of the 2019 novel coronavirus (COVID-19). *J Epidemiol Community Health* (2020) doi:10.1136/jech-2020-214051.

6. Weekly statistics for NHS Test and Trace (England): 3 December to 9 December. *GOV.UK* https://www.gov.uk/government/publications/nhs-test-and-trace-england-statistics-3-december-to-9-december/weekly-statistics-for-nhs-test-and-trace-england-3-december-to-9-december.

7. When to self-isolate and what to do - Coronavirus (COVID-19). *nhs.uk* https://www.nhs.uk/conditions/coronavirus-covid-19/self-isolation-and-treatment/when-to-self-isolate-and-what-to-do/ (2020).

8. If you’re told to self-isolate by NHS Test and Trace. *nhs.uk* https://www.nhs.uk/conditions/coronavirus-covid-19/self-isolation-and-treatment/if-youre-told-to-self-isolate-by-nhs-test-and-trace-or-the-covid-19-app/ (2021).
